# What differs former, light and heavy smokers? Evidence from a post-conflict setting

**DOI:** 10.4314/ahs.v21i1.16

**Published:** 2021-03

**Authors:** Tatjana Gazibara, Marija Milic, Milan Parlic, Jasmina Stevanovic, Nebojsa Mitic, Gorica Maric, Darija Kisic Tepavcevic, Tatjana Pekmezovic

**Affiliations:** 1 Institute of Epidemiology, Faculty of Medicine, University of Belgrade, Belgrade, Serbia; 2 Department of Epidemiology, Faculty of Medicine, University of Pristina temporarily settled in Kosovska Mitrovica, Kosovska Mitrovica, Kosovo, Serbia

**Keywords:** Students, smoking, tobacco, prevention

## Abstract

**Background:**

Evidence suggests that people who live in regions affected by the armed conflict are more likely to smoke.

**Objective:**

The purpose of this study was to assess factors associated with smoking status in a sample of students in the northern Kosovo province.

**Materials and methods:**

A total of 514 students enrolled in University in Kosovska Mitrovica, Kosovo, were recruited between April to June 2015 at Student Public Health Center during mandatory health checks. Participants filled in socio-demographic and behavioral questionnaire and Beck Depression Inventory (BDI). Based on responses about smoking, students were categorized in non-smokers, former smokers, light smokers (1–13 cigarettes/day) and heavy smokers (> 13 cigarettes/day).

**Results:**

Of 514 students, 116 (22.6%) classified themselves as smokers. Higher education level of fathers (Odds ratio [OR]=2.89, 95% confidence interval [CI] 1.30–6.44, p=0.009), not living with smokers (OR=0.42, 95%CI 0.15–0.97, p=0.017) and longer exposure to second hand smoke (OR=1.07, 95%CI 1.01–1.13, p=0.036) was associated with former smoking. Studying medical and natural sciences (OR=2.07, 95%CI 1.05–4.18, p=0.040), consuming alcohol (OR=2.98, 95%CI 1.19–10.03, p=0.020), living with smokers (OR=2.88, 95%CI 1.49–5.56, p=0.002), longer exposure to second hand smoke (OR=1.06, 95%CI 1.01–1.11, p=0.019) and having a more intense depressive symptoms (OR=1.08, 95%CI 1.03–1.13, p=0.002) was associated with light smoking. Being male (OR=0.22, 95%CI 0.07–0.41, p=0.001), older (OR=1.47, 95%CI 1.21–1.78, p=0.001), living with smokers (OR=3.78, 95%CI 1.69–8.07, p=0.001), longer daily exposure to second-hand smoke (OR=1.10, 95%CI 1.04–1.16, p=0.001), and having more severe depressive symptoms (OR=1.12, 95%CI 1.07–1.18, p=0.001) were associated with heavy smoking.

**Conclusion:**

Smoking prevention and cessation programs should include the entire community, because exposure to environmental second hand smoke may facilitate initiation and more intense smoking. Screening of student smokers for depression should be prioritized in the process of rebuilding the framework for primary and secondary prevention in the post-conflict period.

## Introduction

Tobacco use remains the leading cause of preventable morbidity and mortality worldwide 1, 2. At a global level, estimates suggest that tobacco smoking can be attributed to 7.1 million deaths and 182 million disability-adjusted life years 2. In Serbia in 2017, tobacco smoking was largely attributed to disease-specific mortality from lung cancer (77.4%), stroke (33.2%) and ischemic heart disease (23.9%) 3. According to last national health survey, the prevalence of smoking in the Republic of Serbia was approximately 35% among persons aged 15 years and older, while among persons aged 18–64 years was as high as 41.2% 4.

Previous studies suggested that college students were not fully aware of harmful effects of tobacco smoking 5, 6. College students also had a tendency to use a wide range of definitions as to a “smoker” This, in turn, affects their beliefs, motivations for smoking cessation and barriers to quit smoking 7. The problem of tobacco smoking among college/university students has important public health implications. Specifically, this population is expected to be a major task force in the job market in the near future. However, due to the increased risk of developing various chronic diseases as a result of tobacco smoking 3, this health behavior in early adulthood may set the unfavorable health trajectories later in life.

Following the ethnic conflict between Serbs and Albanians in 1999, the Serbian population in Kosovo remains an ethnic minority, with approximately 70,000 persons residing in northern Kosovo and 85,000 persons residing in southern enclaves (around 9.2% of the entire population of Kosovo). The ethnic tensions and occasional conflicts are ongoing between the two ethnic groups, especially in the town of Kosovska Mitrovica 8, 9. Kosovska Mitrovica is situated on the demarcation line between the two ethnic communities and is physically divided by the river Ibar to a northern Serbian part and a southern Albanian part. Foreign military is still present in the Kosovo province as the basic infrastructure is being restored. Institutions at primary healthcare level play an important role in efforts to establish a health care delivery framework to match the degree of activities before the conflict.

Compelling data have shown that people living in regions affected by the armed conflict are more likely to smoke 10 compared to persons living in non-conflict areas. Because previous population health surveys did not include Serbs residing in Kosovo 4 it is difficult to estimate the prevalence of smoking in this particular region. We hypothesized that the prevalence of tobacco smoking among Serbian students in northern Kosovo is higher compared to their peers who study in the remainder of the Republic of Serbia. Identification of factors that contribute to smoking in a university student population is a necessary element to optimize smoking prevention in young adults.

The objectives of this study were to estimate the prevalence of tobacco smoking among Serbian students in the northern Kosovo province as well as to assess factors associated with current smoking status. Data obtained in this study aim at providing the groundwork to estimate the size of the student population who smoke as well as to understand specific behavioral patterns of students smokers in order to address smoking prevention and health care needs of students in the post-conflict period.

## Material and methods

### Participants and setting

Participants in this cross-sectional study were undergraduate students from the University of Pristina temporarily seated in Kosovska Mitrovica (University in Kosovska Mitrovica), northern Kosovo. The University consists of 10 schools divided in four branches: social sciences and humanities, medical sciences, natural sciences and mathematics, and technology and engineering sciences. Currently around 8,000 students are registered at the University. Students were recruited from April to June 2015 at the only Student Public Health Center in Kosovska Mitrovica. Because regular annual health check-ups are mandatory for all the students at the University, this primary-health care facility was suitable for selection of a representative sample of students. Sampling was based on convenience. A total of 521 students were invited to participate in the study. The number of recruited students represents approximately 6.4% of all students at the University in Kosovska Mitrovica. Participation was voluntary and anonymous. Prior to enrollment, students provided informed consent for participation. This study was approved by the Ethics Committee of the University in Kosovska Mitrovica (Approval no. 01-503, issued on April 2, 2015).

### Study instruments

We collected data for this study using a general questionnaire and Beck Depression Inventory. The students filled in the questionnaires independently after their scheduled health checks. The room designated for filling in the questionnaires was adjacent to the physician's office and investigators were at student's disposal for potential questions and clarifications.

The general questionnaire was based on a similar instrument used in previous research about health-related quality of life among students at the University of Belgrade 11. The general questionnaire examined socio-demographic characteristics of the participants and their health behaviors including exposure to environmental secondhand smoke.

Socio-demographic characteristics comprised participants' age, gender, region of origin, type of current residence [with parents/student dormitory/rented apartment/other], parental education level [primary, secondary, university], household monthly income, having own income, type of faculty [social sciences and humanities/medical sciences/nature sciences and mathematics/technology and engineering sciences] and grade point average.

Health behaviors comprised smoking, alcohol intake and physical activity. Smoking was defined as smoking of burning tobacco encased in a cigarette. Alcohol consumption was related to drinking of alcoholic beverages on any occasion. Physical activity was defined as moderate activities for at least 10 minutes at a time, such as brisk walking, cycling, swimming, or any other activity that causes some increase in breathing or heart rate.

Environmental secondhand smoke exposure was examined by asking the students whether they lived with a smoker and whether they were exposed to smoke when other people around them smoke in their presence. In case the students responded that they were exposed to environmental second hand smoke, they were asked to write in the estimated duration of daily exposure.

Additionally, students were asked whether they had chronic illnesses. A list of 8 most common chronic illnesses was provided, based on the previous characterization of most common chronic diseases in students population 12. If none of the listed chronic illnesses corresponded to student's health condition, they were asked to fill in the space assigned for an illness that was not previously mentioned in the list.

The Beck Depression Inventory (BDI) was used to explore feelings and attitudes related to general depressive status 13. It is a one-dimensional scale consisted of 21 items. Answers were graded on a four-point scale from 0 to 3. The total BDI score was obtained as the sum of ratings for each item. The total BDI score ranged from 0 to 63, with higher values denoting presence of more severe depressive symptoms. The psychometric characteristics of the Serbian version of BDI were previously examined 14. The results showed the Cronbach's alpha coefficient of 0.87 and test-retest repeatability of 0.63, suggesting adequate psychometric properties 14.

### Observed outcome

The main outcome of this study was the current smoking status. Students were asked to classify themselves as current smokers, former smokers or non-smokers. Students who reported smoking at least 100 cigarettes in their lifetime and who, at the time of survey, smoked every day or some days were classified as current smokers 15. Students who reported smoking at least 100 cigarettes in their lifetime and who, at the time of the survey, did not smoke at all were defined as former smoker^15^. Non-smokers were those individuals who did not classify themselves in either of the two previously defined groups. Students who smoked were asked to provide data about the quantity of cigarettes smoked per day. Based on the mean number of cigarettes smoked per day in the total study sample, smokers were divided in two categories: “light smokers” (1–13 cigarettes/day) and “heavy smokers” (> 13 cigarettes/day). In this way, we were able to classify all students based on their reported smoking status and number of cigarettes smoked per day on: non-smoker, former smokers, light smokers and heavy smokers. Characteristics of former smokers, light smokers and heavy smokers were compared to non-smokers as the referent category.

### Explanatory variables

We examined variables pertaining to socio-demographics and health behaviours as potential factors associated with former, light and heavy smoking. Age was reported in years. Gender was observed as binary (male vs. female).

The territory of Kosovo has been disputed between Albanians and Serbs. As a result, most Serbs living in Kosovo reside in the northern part of Kosovo, others remain in the southern enclaves surrounded by the territory predominantly settled by ethnic Albanians. Because the freedom of movement in the southern enclaves is largely limited, living circumstances in northern Kosovo and southern enclaves differ. In addition, some students who study in Kosovska Mitrovica come from the remainder of the Republic of Serbia. Because of this, the variable ‘region of origin’ was classified as Serbia/North Kosovo/Southern enclaves.

Current place of residence was dichotomized based on whether students lived with their nuclear family (with parents vs. student dormitory/rented apartment/other). Parental education level for both parents was dichotomized (university level [>12 years] vs. primary and secondary education [≤12 years]). Type of faculty was dichotomized based on similarity of study disciplines (medical and natural science vs. social and engineering science). Students reported household monthly income in Serbian dinars, which was later onverted to Euros. Having own income because of doing paid work was classified as binary variable (yes vs. no).

Grade point average was based on the average grade obtained until the time of survey. The possible range of grades at the University in Kosovska Mitrovica was from 6 (pass) to 10 (excellent). Grade point average was calculated as the sum of all final grades obtained on previously passed exams divided by the number of exams passed. Therefore, the grade point average ranged from 6.0 (as minimum) to 10.0 (as maximum).

Alcohol intake and physical activity were observed as binary variables (yes vs. no). Living with smoker(s) was observed as a binary variable (yes vs. no). Daily exposure to environmental second hand smoke was calculated in hours (range 0–24). Based on students' responses about having chronic diseases, we systematized various chronic diseases to a binary variable (yes vs. no). The BDI score was calculated as the sum of all ratings for 21 questions and was observed as a continuous variable.

### Data analysis

Prevalence of smoking was expressed as percentage. Difference in continuous variables was assessed by Kruskal-Wallis test. Mann-Whitney test was used to locate differences between the multiple groups. Chi square test was used to assess differences in categorical variables. To assess factors associated with smoking status, we performed multinomial logistic regression analysis. Variables with more than 20% of missing data were excluded from the analysis. First we ran univariate regression, to estimate crude association between the explanatory variables with smoking categories. The dependent variable in the models was smoking status (non-smoker vs. former smoker/light smoker/heavy smoker). All variables univariately statistically significant entered the final multivariate multinomial logistic regression model. Probability level of ≤ 0.05 was considered statistically significant. The Statistical Package for Social Sciences (SPSS) 20.0 statistical software package (SPSS Inc, Chicago, IL, USA) was used to perform the analysis.

## Results

### Description of the study group

Of 521 invited students, the study sample included 514 participants (response rate 98.6%). A total of 116 (22.6%) students classified themselves as current smokers (23.1% were males and 22.1% were females). There were 33 (6.4%) former smokers. The average age at smoking initiation was 17.3 ± 1.9 years (maximum age range at smoking initiation 12–23 years). The mean duration of smoking was 3.5 ± 2.2 years. Students smoked on average 13.9 ± 9.3 cigarettes per day (range 1–70 cigarettes). Based on the quantity of cigarettes smoked per day (mean 13 cigarettes/day), there were 63 (54.3%) light smokers and 53 (45.7%) heavy smokers. Of 53 heavy smokers, 32 (60.4%) reported smoking 20 or more cigarettes per day.

Basic demographic characteristics according to smoking status are presented in Table 1. The variable ‘region of origin’ included 40% of missing data and was therefore excluded from further analyses. The four student groups differed in terms of age, gender, living arrangements, father's education level, having own income, type of faculty, alcohol intake, living with smokers, exposure to second hand smoke, having chronic diseases and BDI scores. Majority of light and heavy smokers lived with smokers. Most students, regrardless of their smoking status, were exposed to second hand smoke. The average length of daily exposure to second hand smoke was 6 hours. The students who were classified as “heavy smokers” were more often exposed to daily second-hand smoke for more than 6 hours (Table 1).

**Table 1:** Characteristics of students at the University in Kosovska Mitrovica according to smoking (n = 514)

Variable	Non- smokers n = 3 65	Former smokers n = 33	Light smokers n = 63	Heavy smokers n = 53	P for difference
Age	21.0 (1.8)	20.7 (1.6)	21.1 (1.3)	21.6 (3.1)	0.011
Gender - male - female	162 (44.4) 203 (55.6)	21 (63.6) 12 (36.4)	24 (38.1) 39 (31.9)	31 (58.5) 22 (41.5)	0.024
Region of origin - Serbia - North of Kosovo - Southern enclaves - *Missing*	108 (49.8) 60 (27.6) 49 (22.6) *148 (40.5)*	10 (55.6) 5 (27.8) 3 (16.7) *15 (45.4)*	16 (45.7) 9 (25.8) 10 (28.6) *28 (44.4)*	19 (50.2) 9 (27.2) 7 (22.6) *18 (34.0)*	0.967
Type of current residence - home (with parents) - students' dormitory - alone (rented apartment) - other	106 (29) 142 (38.9) 110 (30.1) 7 (1.9)	4 (12.1) 9 (27.3) 18 (54.5) 2 (6.1)	17 (27.0) 22 (34.9) 20 (31.7) 4 (6.3)	13 (24.5) 16 (30.2) 22 (41.5) 2 (3.8)	0.048
Mother's education level - higher - other	87 (23.8) 278 (76.2)	9 (27.3) 24 (72.7)	15 (23.8) 48 (16.2)	19 (35.8) 34 (64.2)	0.298
Father's education level - higher - other	120 (39.2) 245 (67.1)	19 (57.6) 14 (42.4)	15 (23.8) 48 (76.2)	20 (37.7) 33 (62.3)	0.009
Family monthly income (Eur)	507 (348)	427 (140)	621 (813)	705 (532)	0.292
Having own income - yes - no	155 (42.5) 210 (57.5)	8 (24.2) 25 (75.8)	18 (28.6) 45 (71.4)	12 (22.6) 41 (77.4)	0.004
Type of faculty - Medical science - Social science and humanities - Natural sciences and math - Technology and engineering	175 (47.9) 105 (28.8) 8 (2.2) 77 (21.1)	18 (54.5) 7 (21.2) 3 (9.1) 5 (15.2)	22 (34.9) 15 (23.8) 1 (1.6) 25 (39.7)	23 (43.4) 16 (30.2) 2 (3.8) 12 (22.6)	0.030
Grade point average*	7.8 (0.9)	7.6 (0.6)	7.9 (0.9)	7.7 (0.7)	0.540
Alcohol use - yes - no	138 (37.8) 227 (62.2)	5 (15.2) 28 (84.8)	10 (15.9) 53 (84.1)	10 (18.9) 43 (81.1)	0.001
Physical activity - yes - no	62 (17.0) 303 (83.0)	9 (27.3) 24 (72.7)	16 (25.4) 47 (74.6)	10 (18.9) 43 (81.1)	0.249
Living with smokers - yes - no	145 (39.7) 220 (60.3)	21 (63.6) 12 (36.4)	44 (69.8) 19 (30.2)	37 (69.8) 16 (30.2)	0.001
Daily exposure to indoor second hand smoke - yes - no	213 (58.4) 152 (41.6)	26 (78.8) 7 (21.2)	50 (79.4) 13 (20.6)	39 (73.6) 14 (26.4)	0.001
Duration of daily exposure to second hand smoke - 0 - 1–5 hours - 6–12 hours - >12 hours	152 (41.6) 171 (46.9) 24 (6.6) 18 (4.9)	7 (21.2) 18 (54.6) 4 (12.1) 4 (12.1)	13 (20.6) 32 (50.8) 10 (15.9) 8 (12.7)	14 (26.4) 20 (37.8) 7 (13.3) 12 (22.5)	0.001
Having chronic diseases - yes - no	336 (92.1) 29 (7.9)	29 (87.9) 4 (12.1)	52 (82.5) 11 (17.5)	42 (79.2) 11 (20.8)	0.009
BDI score	2.6 (840)	3.2 (4.6)	4.2 (5.1)	9.1 (11.6)	0.001

Legend: BDI-Beck Depression Inventory; normally distributed values are presented as mean (standard deviation). not normally distributed values are presented as median (interquartile range); non-parametric variables are presented as count (percentage)

* grading system ranges from 6 as minimum (lowest passing grade) to 10 as maximum (highest passing grade)

### Results of univariate analysis

Age, gender, current residence, fathers' education level, having own income, type of faculty, alcohol intake, living with smokers, exposure to second hand smoke, having chronic diseases and BDI were univariately associated with either former, light or heavy smoking (Table 2). Education of mothers, household monthly income, grade point average and physical activity were not associated with the observed smoking categories.

**Table 2:** Factors associated with smoking among of students at the University in Kosovska Mitrovica: results of univariate multinomial logistic regression analysis (non-smokers as referent group)

Variable	Former smokers n = 33			Light smokers n = 63			Heavy smokers n = 53		
	OR	95% CI	p	OR	95% CI	p	OR	95% CI	p
Age	0.97	0.75–1.23	0.775	1.01	0.84–1.23	0.946	1.33	1.14–1.55	0.001
Gender female vs. male	2.19	1.05–4.59	0.037	0.77	0.45–1.40	0.353	1.77	0.99–3.17	0.056
Current residence with parents vs. other	0.35	0.12–1.02	0.055	2.54	1.19–5.42	0.016	2.94	1.36–6.35	0.006
Mother's education level higher vs. other	1.44	0.59–3.50	0.421	0.79	0.39–1.60	0.507	0.56	0.28–1.12	0.100
Father's education level higher vs. other	0.32	014–0.72	0.006	1.82	0.91–3.65	0.093	1.11	0.56–2.20	0.771
Household monthly income	n/a			n/a			n/a		
Having own income yes vs. no	0.43	0.19–0.99	0.046	0.54	0.30–0.97	0.040	0.40	0.20–0.78	0.007
Type of faculty social and engineering science vs. medical and natural science	1.74	0.83–23.64	0.141	0.57	0.33–0.99	0.047	0.89	0.50–1.58	0.687
Grade point average	0.77	0.44–1.46	0.376	1.14	0.79–1.66	0.487	0.84	0.57–1.24	0.368
Alcohol use yes vs. no	0.29	0.11–0.78	0.014	0.31	0.15–0.63	0.001	0.38	0.19–0.79	0.009
Physical activity yes vs. no	1.83	0.81–4.13	0.114	1.66	0.89–3.12	0.113	1.14	0.54–2.38	0.735
Living with smokers Yes vs. no	2.66	1.27–15.56	0.010	3.51	1.97–6.26	0.001	3.51	1.88–6.54	0.001
Daily exposure to second hand smoke (hours)	1.08	1.03–1.14	0.002	1.10	1.06–1.14	0.001	1.11	1.07–1.16	0.001
Having chronic diseases yes vs. no	1.44	0.46–4.46	0.531	2.53	1.19–5.42	0.016	2.94	1.36–6.35	0.006
BDI score	1.14	0.98–1.12	0.146	1.08	1.04–1.14	0.001	1.12	1.08–1.17	0.001

Legend: OR - odds ratio; CI - confidence interval; p – probability level; BDI - Beck Depression Inventory; vs. - versus.

### Results of multivariate analysis

Former smokers were more likely to have highly educated fathers', not live with smokers and be exposed to second hand smoke for longer periods of time (Table 3). Studying medical and natural sciences, consumption of alcohol, living with smokers, longer daily exposure to second hand smoke and having more intense depressive symptoms was associated with light smoking (Table 3). Heavy smokers were more likely to be males, older, living with smokers and be exposed to daily second hand smoke for longer periods. Heavy smokers were also more likely to have more severe depressive symptoms (Table 3).

**Table 3:** Factors associated with smoking status among students at the University in Kosovska Mitrovica: results of multiple multinomial logistic regression analysis (non-smokers as referent group)

Variable	Former smokers n = 33			Light smokers n = 63			Heavy smokers n = 53		
	OR	95% CI	p	OR	95% CI	p	OR	95% CI	p
Age	0.91	0.68–1.22	0.527	1.02	0.83–1.26	0.815	1.47	1.21–1.78	**0.001**
Gender female vs. male	1.96	0.85–4.53	0.116	0.97	0.51–1.83	0.920	0.22	0.07–0.41	**0.001**
Current residence with parents vs. other	2.31	0.74–7.22	0.150	1.23	0.62–2.45	0.560	1.15	0.52–2.56	0.733
Father's education level higher vs. other	2.89	1.30–6.44	**0.009**	0.89	0.45–1.77	0.737	2.34	0.86–7.39	0.112
Having own income yes vs. no	0.80	0.33–1.96	0.625	0.78	0.40–1.52	0.467	1.37	0.54–6.73	0.111
Type of faculty medical and natural science vs. social and engineering science	1.11	0.48–2.56	0.807	2.07	1.05–4.18	**0.040**	0.83	0.40–1.72	0.619
Alcohol use yes vs. no	0.36	0.13–1.01	0.052	2.98	1.19–10.03	**0.020**	1.19	0.36–3.25	0.284
Living with smokers Yes vs. no	0.42	0.15–0.97	**0.017**	2.88	1.49–5.56	**0.002**	3.78	1.69–8.07	**0.001**
Daily exposure to indoor second hand smoke (hours)	1.07	1.01–1.13	**0.036**	1.06	1.01–1.11	**0.019**	1.10	1.04–1.16	**0.001**
Having chronic diseases yes vs. no	0.68	0.17–2.61	0.570	0.52	0.20–1.35	0.180	0.47	0.17–1.34	0.160
BDI score	1.03	0.97–1.11	0.327	1.08	1.03–1.13	**0.002**	1.12	1.07–1.18	**0.001**

Legend: OR - odds ratio; CI - confidence interval; p - probability level; BDI - Beck Depression Inventory; vs. – versus; bold values denote statistical significance.

## Discussion

### Principal findings

This study found that more than one fifth of students at the University of Kosovska Mitrovica identified themselves as smokers. The prevalence of female smokers was only slightly lower than that among males. We observed that of all smokers, there were more light smokers compared to heavy smokers. Most heavy smokers smoked at least one pack of cigarettes per day. Factors associated with former, light and heavy smoking differed between the three categories. However, compared to non-smokers, former, light and heavy smokers were exposed to environmental second hand smoke more hours per day, suggesting that smoking in public places, on campus and at home might be the main contributor of smoking in this population group. Having highly educated fathers, not living with smokers and longer exposure to second hand smoke was associated with being former smoker. Studying health and natural sciences, consuming alcohol, living with smokers, longer exposure to second hand smoke and having more depressive symptoms was associated with light smoking. Being male, older, living with smokers, longer daily exposure to second hand smoke and having more intense depressive symptoms was associated with heavy smoking.

### Comparison with similar studies

The results of our study supported the hypothesis that the prevalence of smoking among Serbian students in the northern Kosovo province is higher than that in the Republic of Serbia (22.6% vs. 17.9%) 16. In terms of regions affected by the armed conflict, the prevalence observed in this study is similar to that among students who smoked during the crisis in Syria (24.7%) 17. However, at a global level, frequency of student smokers in North Kosovo is comparable to those reported in Hungary or Venezuela 18. The highest prevalence of male student smokers has been previously observed in Portugal (47%), Greece (44%) and Bulgaria (42%) 18. Female smokers were the most prevalent in Spanish and Bulgarian University population (both 46%) 18. The above-mentioned smoking rates have been attributed to cultural factors, which are specific to Southern and Eastern European region 18. Our results suggest that even though Serbian students in northern Kosovo smoke more than their peers in the Republic of Serbia, prevalence of smoking in this area is still lower than in some neighboring countries (such as Greece or Bulgaria) with no history of recent armed conflict.

The definition of being a “heavy smoker” is not consistent in previous studies. For example, Sutfin et al. labeled students who reported smoking of 6–10 cigarettes per day as “heavy smokers” 19, while the same category in the study of Jason et al. 20 was used to define individuals who smoked more than 20 cigarettes. Therefore, the definition of “heavy smoker” in our study is specific to our student sample. Sutfin et al. 19 suggested that, in some populations, students who smoked belonged to a more heterogeneous group. In fact, aside from heavy smokers, the authors identified subgroups such as “social smokers” who usually smoked on weekends, “puffers” who smoked 1–2 days per month, and “no-context” smokers who smoked, on average, 2–5 cigarettes every other day 19. These categories may comprise a group of “light” or “intermittent” smokers 21, in which the individuals do not smoke every day. However, “light smokers” in our set of participants were, indeed, daily smokers, but less intensive compared to “heavy smokers”. This classification contrasts some US studies where many students smoke, but daily smoking is not frequent 22, 23.

Alcohol intake has been previously associated with “moderate” and “social” smoking patterns 19. A qualitative study documented that, through socializing in college, students who drink alcohol usually initiate tobacco use 24. Alcohol-smoking dyad is suggested to be part of the individualization process, particularly among the college freshmen 25. Given that this characteristic was not associated with being a “heavy smoker” in our study sample, we may consider that the social environment plays a more important role in acceptance of smoking. It is intriguing that students in medical sciences were more likely to smoke compared to students in other branches. Practice of smoking stands in contrast to studying health-related disciplines, because future physicians are expected to advocate smoking prevention and smoking cessation. Reasons behind this finding might be more complex, and therefore, difficult to explain without further qualitative analysis in this subgroup of students. Still, high smoking prevalence among medical students has been observed in the Middle East 26 and Southern Europe 27, which likely reflects smoking patterns in their respective general populations.

Having more depressive symptoms was associated with both light and heavy smoking. This association has been observed earlier among students in the Republic of Serbia 16 as well as worldwide 28, 29. The association between depression and smoking has previously been explained by feeling that the cigarettes are often regarded as a ‘companion’ 29. Thus, the individual establishes a ‘friendship link’ with the smoking process, whereas actual social functioning could be insufficient^29^. It is recommended that students who have depressive symptoms be eligible for interventions to increase social support 29, which, in turn, could have favorable effect on smoking cessation.

Aside from the depressive symptoms, heavy smokers in our sample were more likely to be males and older, live with smokers and be exposed to daily second hand smoke for prolonged periods. Our findings are in line with reports from the National Health Survey in 2013, where the prevalence of smoking among men in the Republic of Serbia has consistently been higher than that in women over the past two decades 4. These data suggest that one in five males smoked more than 20 cigarettes daily, compared to one in nine women 4, whereas in our sample of students, most heavy smokers smoked 20 or more cigarettes per day. It has been reported that 83% of college students are exposed to second-hand smoke 30, which matches our findings. Domestic aspect of smoking appears to be of major influence on heavy smokers in our study. This means that a strict ban on smoking in dormitories, university facilities and public spaces needs to be put forwards.

Our results are consistent with previous reports 16, 31, where the effect of second hand smoke exposure in the households and in public places (bars, restaurants, cars, etc.) and living with smokers have been observed as contributors to smoking among students. These findings suggest that behavioral patterns are shared with family members are mirrored across the age groups. This feature is particularly relevant, as factor associated with smoking cessation (i.e. being a former smoker) among the students in our study was the absence of smokers in the household. Thus, overall smoking prevention and tobacco control need to be improved, while smoking in public places should be sanctioned according to the Law on Protection of the Population from Exposure to Tobacco Smoke 32 passed in 2010 in the Republic of Serbia.

## Limitations and strengths of the study

Limitations of this study include reporting bias, due to self-reported smoking, alcohol use and physical activity. Therefore, it is possible that smoking prevalence could have been under-reported. Although students completed the questionnaire independently, we cannot exclude discussions about the survey outside of the physician's office in the corridors of the Student Public Health Center. Therefore, the study might be open to a social acceptability bias, as students who completed their health check could have recommended the other students who were waiting for their appointment to fill in the questionnaire. However, using another strategy of recruitment outside the Student Public Health Center would have introduced a selection bias, because health checks are mandatory for all students enrolled in the University of Kosovska Mitrovica. While we intended to analyze the variable ‘region of origin’, there was a large proportion of missing data. For this reason, we were not able to draw meaningful analysis of this particular variable. Potential explanation for missing information might be that students fear to report their region of origin, regardless of anonymity of research. Because of cross-sectional design, we are not entirely certain about the direction of the association, and as such, we could only suggest potential inference and not determine causality. Participants in this study constitute a specific population. As a result, our observations have limited generalizability to areas not affected by armed conflict.

This study is the first to examine smoking patterns among students in the northern Kosovo province. Previous studies at a national-level did not include Serbian population living in Kosovo due to complex socio-political circumstances. Therefore, until now, health behaviors such as smoking, in this population were largely unknown. Another strength of this study is that a representative sample of students from the University of Kosovska Mitorovica was recruited, because students are required by University bylaw to present at health check-up before the end of the school year. Failure to undergo periodic health checks would disqualify the students to take the exams in the summer exam session. Recruitment of the study sample from the Student Public Health Center allowed for selection bias to be minimized.

## Importance of the study for public health

Findings from this study suggest that there is a need for public health interventions specifically tailored for students, with the goal to reduce or prevent smoking. Moreover, because public spaces are not free from smoke and exposure to environmental smoke likely influences the acceptability and social norms in and out of the school campus. These norms can, therefore, favor the initiation and increase in smoking intensity among students. To address the issue of smoking in public spaces, the community as a whole should be included in smoking prevention programs along with the enforcement of the Law on Protection of the Population from Exposure to Tobacco Smoke 32. The Law also needs to be implemented strictly and without exceptions.

The Student Public Health Center could be the principal institution for prevention of smoking among students in Kosovska Mitrovica and for counseling about smoking cessation, because students are already required to frequent the Center for period health checkups. In management of health conditions among University students, use of tobacco and tobacco cessation has been addressed 33, as this specific exposure might complicate management of chronic diseases or worsen the clinical symptoms, particularly that of respiratory diseases. The Center could organize workshops with experts from and outside of the local community to discuss long-term effects of smoking on individual physical 34 and mental health 35 as well as that in the offspring 36. Similarly, use of digital technology, such as smartphone apps 37 or online discussion forums could be a good tool to encourage smoking cessation among young adults. The Center could set up an online website for counseling and support to students who wish to quit smoking.

## Possibilities for future research in the field

Although one-time cross-sectional data collection can provide orientation about point prevalence of smoking, this study could be further elaborated into repeated cross-sectional measurements over several years or decades. Because cross-sectional study design limits causal inference between the explanatory variables and the outcome, it is recommended to follow the student cohorts longitudinally. For example, the cohort of students in the first year at the University could be followed until their graduation to estimate the dynamics of potential change in the prevalence of smoking. Qualitative studies in focus groups or in-depth interviews may help to better understand the motivators behind smoking initiation and more intense smoking. To estimate the effectiveness of smoking prevention and smoking cessation programs, measurement before and after intervetions could provide a valuable insight into the degree of change of health behaviors.

## Conclusion

Our findings highlight that factors associated with former, light and heavy smoking among students who live in a post-conflict region differ to a certain extent. We have observed that longer daily exposure to second hand smoke was the only contributor that was consistently associated with all the three subgroups of students. Living with smoker was common for being light or heavy smoker. In addition, heavy smokers were also more likely to have depressive symptoms, similar to light smokers. These findings suggest that the primary health care sector and students' health care service in the northern Kosovo province should prioritize promotion of smoking cessation as a long-term public health strategy. Smoking prevention and cessation programs should include the entire community, because exposure to environmental second hand smoke may facilitate initiation and more intense smoking. In a similar vein, periodical health checks of students should include screening for depression, especially among those students who identify themselves as smokers.
